# Direct Identification of On-Bead Peptides Using Surface-Enhanced Raman Spectroscopic Barcoding System for High-Throughput Bioanalysis

**DOI:** 10.1038/srep10144

**Published:** 2015-05-28

**Authors:** Homan Kang, Sinyoung Jeong, Yul Koh, Myeong Geun Cha, Jin-Kyoung Yang, San Kyeong, Jaehi Kim, Seon-Yeong Kwak, Hye-Jin Chang, Hyunmi Lee, Cheolhwan Jeong, Jong-Ho Kim, Bong-Hyun Jun, Yong-Kweon Kim, Dae Hong Jeong, Yoon-Sik Lee

**Affiliations:** 1 Interdisciplinary Program in Nano-Science and Technology, Seoul National University, Seoul 151-744, Republic of Korea; 2 Department of Chemistry Education, Seoul National University, Seoul 151-744, Republic of Korea; 3 School of Electrical Engineering and Computer Science, Seoul National University, Seoul 151-744, Republic of Korea; 4 School of Chemical and Biological Engineering, Seoul National University, Seoul 151-744, Republic of Korea; 5 Department of Chemical Engineering, Hanyang University, Ansan, 426-791, Republic of Korea; 6 Department of Bioscience and Biotechnology, Konkuk University, Seoul 143-701, Republic of Korea

## Abstract

Recently, preparation and screening of compound libraries remain one of the most challenging tasks in drug discovery, biomarker detection, and biomolecular profiling processes. So far, several distinct encoding/decoding methods such as chemical encoding, graphical encoding, and optical encoding have been reported to identify those libraries. In this paper, a simple and efficient surface-enhanced Raman spectroscopic (SERS) barcoding method using highly sensitive SERS nanoparticles (SERS ID) is presented. The 44 kinds of SERS IDs were able to generate simple codes and could possibly generate more than one million kinds of codes by incorporating combinations of different SERS IDs. The barcoding method exhibited high stability and reliability under bioassay conditions. The SERS ID encoding based screening platform can identify the peptide ligand on the bead and also quantify its binding affinity for specific protein. We believe that our SERS barcoding technology is a promising method in the screening of one-bead-one-compound (OBOC) libraries for drug discovery.

The preparation and screening of large compound libraries remains one of the most challenging tasks in drug development, as well as in the detection of multiplexed disease-biomarker and biomolecular profiling^1^,^2^. Several distinct encoding methods, including chemical, graphical, and optical encoding, have been reported thus far on microcarriers in preparation of large compound library. In the chemical encoding, the microcarriers are encoded by attaching detectable molecular tags such as oligonucleotides^3^,^4^, haloaryls^5^, trityls^6^, and fluorescent dyes^7^. Chemical encoding method, however, needs compatible tags and tag synthesis reactions that may cause artifacts against the library synthesis. In addition, this method requires laborious and expensive procedures to analyze molecular tags to identify lead compounds^8^. Alternative graphical and optical encoding methods using pre-encoded microparticles^7^,^9^,^10^,^11^ have been reported, which can decode directly without requiring further chemical process^12^,^13^,^14^,^15^,^16^,^17^,^18^,^19^,^20^,^21^,^22^,^23^. In the graphical encoding, microparticles are distinguished based on their shapes or internal patterns of optical elements^24^, which can be modulated using lithographic micro-fabrication processes^25^,^26^ or selective photobleaching/curing^17^,^27^. Doyle and co-workers have presented a method based on continuous-flow lithography that combines dot-patterned particle synthesis and probe conjugation into a single process to generate multifunctional encoded particles bearing over a million unique codes^19^. Optical encodings commonly rely on specific color or spectroscopic information of light emitted from several optical materials such as fluorescence dyes^7^,^23^,^28^,^29^, quantum dots (QDs)^12^,^20^, photonic structures^14^,^30^, and Raman tags^9^,^10^,^11^,^31^. Nie and co-workers have reported the optical encoding technology based on QDs entrapped into polystyrene microbeads by solvent swelling methods^12^. In theory, six colors at six different intensities would yield around 40,000 different codes, but in practice, overlap between the different intensities is a major limitation^8^. In addition, these graphical and optical encoding methods present several drawbacks: (i) they lack massive parallel coding to produce “pre-encoded microcarriers”; (ii) encoded microcarriers, which initially present a non-biocompatible cross-linked polymer environment, must be functionalized for further conjugation of ligands; (iii) a sequential attachment of fully synthesized bio-ligands is required; and (iv) the decoding process is not suitable for automation due to complex codes, which could lead to an ambiguous interpretation, and due to the fact that an orientation of the encoded microcarriers must be determined before the decoding process^8^,^32^,^33^. Developing novel encoding methods, therefore, faces a challenge such as enabling easy encoding on a large-scale in water-compatible microcarriers, and rapid and automatic decoding of each encoded microcarrier.

Recently, a fluorescent silica-based “colloidal barcoding” method as one of the optical encoding has been reported by Trau and co-workers. This barcoding method is the first report for tracking of synthetic path during split-and-mix synthesis by using fluorescent silica nanoparticles (NPs) which contain specific, and identifiable combinations of fluorescent dyes^34^,^35^,^36^,^37^. However, a large number of fluorescence-based codes are impractical due to complex nature of the process, which includes issues with spectral overlapping and with fluorescence resonance energy transfer^13^,^38^. For high-throughput screening of large libraries, it is essential for a novel encoding method to be equipped with a large encoding capacity that confers a reproducible and photostable codes. In this regard, alternative surface-enhanced Raman scattering (SERS) NPs-based colloidal barcoding method has been introduced^31^,^39^. This SERS encoding method was applied during solid-phase peptide synthesis to identify the peptide sequence by encoding each amino acid with the corresponding SERS NPs. The SERS based encoding relies on strong SERS signals exhibiting a narrow bandwidth (<2 nm) without overlapping. For detecting multiple SERS NPs, a single-wavelength excitation is used^40^,^41^,^42^,^43^,^44^,^45^.

Here, we report a more simple and efficient surface-enhanced Raman scattering (SERS)-based encoding which can be utilized for high-throughput screening. In contrast with the previous report, after full sequence of peptide was constructed on polymer beads, the peptides were encoded with combinations of SERS active NPs, termed as SERS nano-identifier (SERS ID). The 44 kinds of SERS IDs were able to generate simple codes and could possibly generate more than one million kinds of codes by incorporating combinations of different SERS IDs. The SERS codes that were generated from a combination of five SERS IDs were decoded successfully and transformed automatically into simple barcodes. Using SERS ID-encoding, we performed a binding assay that was used to screen streptavidin-binding peptides from a one-bead-one-compound (OBOC) library. The library screening platform with SERS ID encoding not only identified the peptide sequences on the beads but also could quantify the binding affinity of a peptide ligand toward specific proteins.

## Results and Discussion

**Figure 1:**
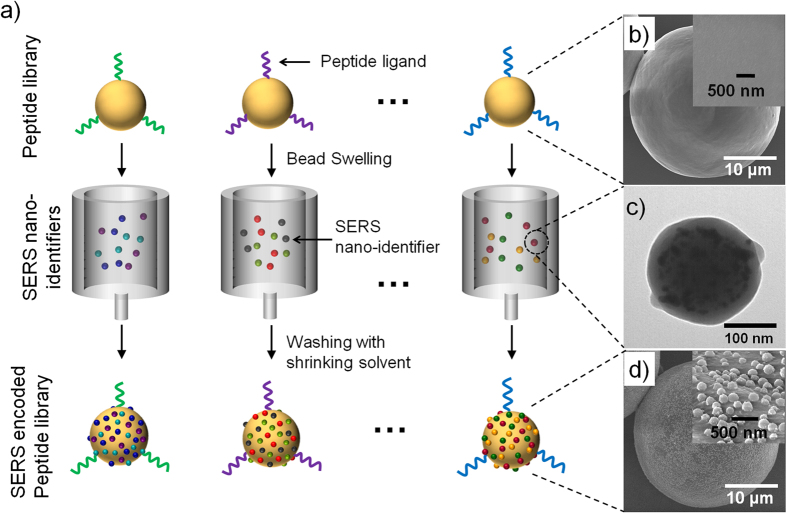
Schematic diagram of the peptide-encoding process with SERS nano-identifiers (SERS IDs) and electron microscopic images at each step. **a**) Peptide-encoding process by attaching SERS IDs. **b**) Field-emission scanning electron microscope (FE-SEM) images of TentaGel (TG) microbeads without encoding (inset: High-magnification image of TG bead surface). **c**) Transmission electron microscope image of SERS ID, consisted of Ag NPs embedded in silica nanosphere. **d**) FE-SEM images of TG beads with SERS encoding (inset: High-magnification image of polymer bead surface bearing SERS IDs).

Figure 1a shows the peptide-encoding strategy. Many sets of peptide sequences were synthesized on commercially available TentaGel (TG) beads (~35 μm). After the synthesis, the TG beads were swollen with NMP solution. The swollen volume of TG bead in NMP was ~2.5 times larger compared to dried TG bead. The swollen TG beads were then mixed with the corresponding SERS ID dispersion for 10 min to confer the SERS codes to the specific peptide-TG beads, which were physically adsorbed on the TG bead surfaces. The TG beads were then washed several times with ethanol, which acted as a shrinking agent, resulting in collapse of polymer chains of TG bead with the SERS IDs. Scanning electron microscope (SEM) image of the peptide-TG beads showed clean and smooth surfaces before SERS encoding (Fig. 1b). However, after the encoding process, the SERS IDs (~200 nm, TEM image is shown in Fig. 1c) were adsorbed and buried halfway onto the TG bead surfaces via solvent-driven swelling and shrinking process, as shown in Fig. 1d (37,000 dots/single bead in average could be loaded). The buried structure indicates that the SERS IDs might be bridging the polymer chains of the TG beads. The formation of a polymer chain bridge with the SERS IDs on the TG beads could potentially enhance the stability of the SERS IDs.

The total number of possible codes (*N*_code_) was determined from the following equation:


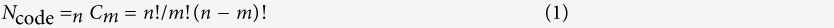


where, *n* is the number of available kinds of SERS IDs, and *m* is the number of selected SERS IDs. As shown in Figure S1, the 44 kinds of SERS IDs were available, based on simple aromatic label compounds. The maximum number of selectable SERS IDs, *m*_*max*_, is estimated to be the maximum number of SERS ID particles attached on a TG bead (*NS*_max_) divided by the least number of one kind of SERS ID particles on the TG bead (*NS*_min_), which provides enough amount of signal for detection. The *NS*_max_ value was determined from the saturation level of SERS IDs when the amount of SERS IDs is increased to a given amount of TG beads. The saturation amount was estimated to be 88 particles on a single TG bead of 9 μm^2^ surface area in one side (Figure S2). It was assumed that the intensity of SERS signal should be 20 counts/s to obtain a detectable signal, which was two times the noise level in the current experimental condition. Thus, the *NS*_min_ value was estimated to be 18 SERS ID particles on a 9 μm^2^ surface area of the TG bead. The estimation was performed on SERS IDs coded with 4-fluorobenzenthiol, denoted as SERS ID_[4FBT]_ with their SERS intensity at 384 cm^−1^. The maximum number of selectable SERS IDs was obtained from the following equation:


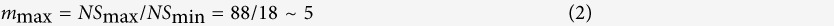


**Figure 2:**
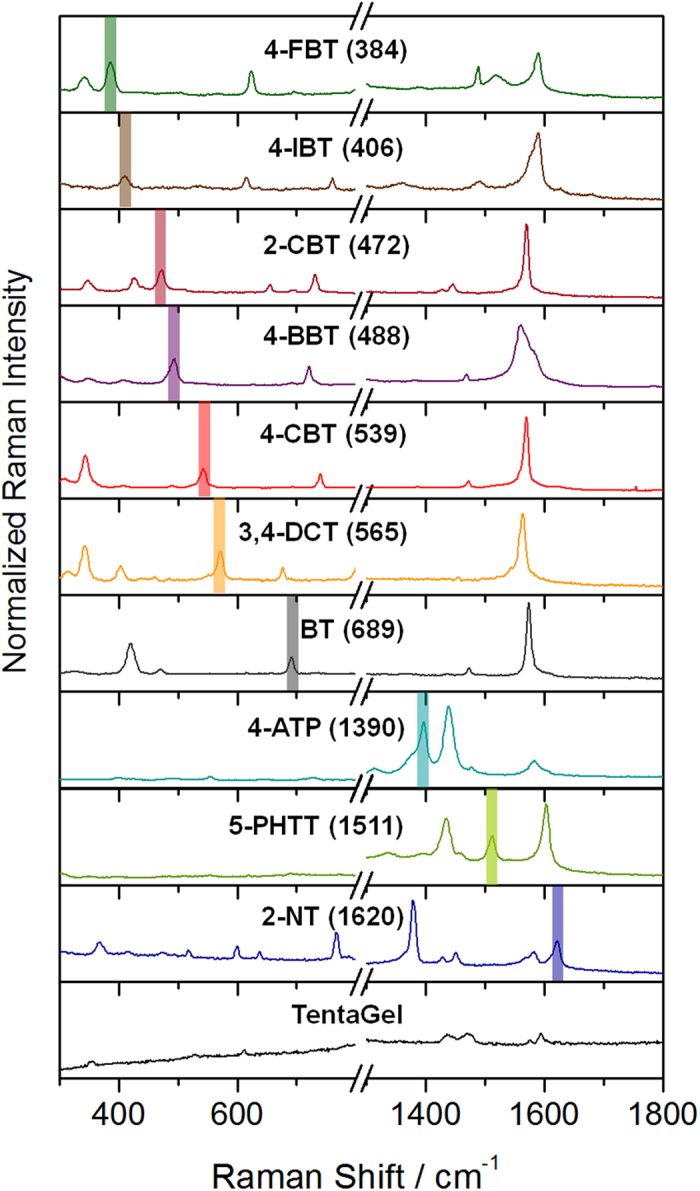
SERS spectra of SERS nano-identifiers (SERS IDs) and TentaGel microbeads. Colored bars indicate representative peaks without spectral overlap.

**Figure 3:**
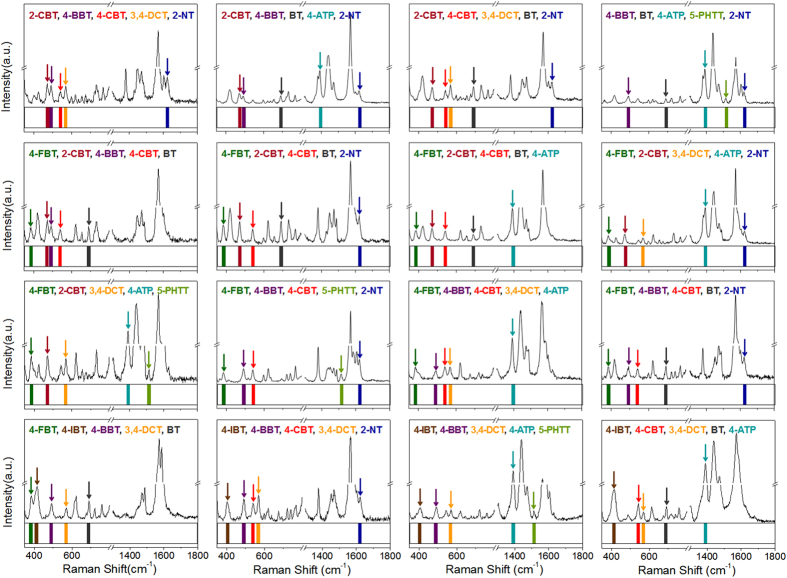
Sixteen representative SERS spectra and their corresponding barcode presentations for TentaGel beads encoded with 5 SERS nano-identifier (SERS ID) combinations.

Based on the estimation, where *n* = 44 and *m* = 5, over one million total codes (*N*_code_ = 1,086,008) could be generated using eq. (1). The large number of total codes is suitable for encoding of a large size compound/peptide library. While optical encoding commonly relies on both the color and intensity of emission signals from more than one luminescent material such as fluorescent dye or quantum dot^12^,^20^,^21^, the SERS ID-based encoding does not require a complex control of intensity for a large number of codes, which is the major advantage. A number of simple aromatics that contain thio, isothiocyanato, azido, or cyano groups can be utilized as a Raman label compound^31^. Figure 2 shows the spectra of 10 types of SERS IDs with a unique representative peak (represented as a colored bar) without any overlap in the same detection window. Each SERS ID coded with different Raman label compounds had the same physical properties (size, shape, and surface) (Figure S3) because each SERS ID has the same silica core and silica shell. Hence, each SERS ID contributed equally to the surface adsorption on the TG beads when a mixture of various SERS IDs was added to the bead surfaces. As shown in Fig. 3, 16 kinds of distinct barcodes could be generated from 5 SERS ID combinations, demonstrating the potential of a large number of optical encodings. The 5 kinds of SERS IDs were shaken together with the TG beads. For the combination of SERS IDs, different ratios were used because the enhancement effect of each Raman label compound is different (details in Methods section). The SERS peaks from SERS-coded beads were then measured by a 532 nm single-excitation source. Based on the results determined by processing algorithms (details of the peak detection algorithms using Matlab® are provided in Methods section), 5 unique representative peaks from each single bead were effectively deconvoluted and automatically converted to barcode presentations (Figure S4). In addition, the 5 codes for SERS IDs-encoded TG bead (encoded with SERS IDs_[4-FBT], [2-CBT], [4-CBT], [BT],_ and _[4-ATP]_) could be clearly read out from a point-by point mapping of a single bead (Figure S5). The TG beads encoded with SERS IDs_[4-FBT], [2-CBT], [4-BBT], [4-CBT],_ and _[BT]_ showed reproducible SERS spectra from each bead (Figure S6). Taken together, the results show that the combination of SERS IDs allowed successful encoding/decoding of TG beads.

**Figure 4:**
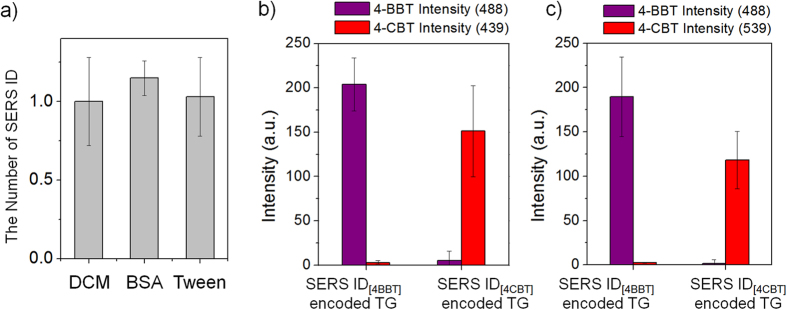
Stability and cross-contamination test of SERS nano-identifier (SERS ID)-encoded TentaGel (TG) beads. **a**) Average number of SERS IDs on the TG beads after treatment with swelling solvents (dichloromethane, DCM), 3% bovine serum albumin (BSA) containing phosphate-buffered saline (PBS, pH 7.0) and 1% Tween 20 containing PBS (pH 7.0). The number was normalized to the total number of SERS IDs on non-treated beads. **b**) Signal intensities of 488 cm^−1^ band (4-BBT) and 539 cm^−1^ band (4-CBT) from SERS spectrum of SERS ID_[4BBT]_- or SERS ID_[4CBT]_-encoded TG beads after treatment with 3% BSA containing PBS (pH 7.0), and **c**) 1% Tween 20 containing PBS (pH 7.0) (The number of measured beads = 6).

The stability of the SERS IDs on the TG beads was assessed in the presence of i) a swelling solvent (dichloromethane), ii) blocking solution (3% bovine serum albumin [BSA] containing phosphate-buffered saline [PBS]), and iii) surfactant containing washing buffer (1% Tween 20 containing PBS). SERS ID-encoded Ac-Gly-TG beads were treated with each solvent for 30 min. After the treatment, the average number of SERS IDs per 9 μm^2^ area of the TG beads was calculated and normalized to the total number of SERS IDs on non-treated beads, where counts were based on SEM images (representative SEM images are shown in Figure S7). As shown in Fig. 4a, the average number of SERS IDs on the TG beads after treating with each condition did not decrease significantly. To evaluate the SERS ID stability further, cross-contamination tests were performed using the mixture of SERS IDs_[4BBT]_-encoded TG beads and SERS ID_[4CBT]_-encoded TG beads. The SERS intensities of 4-BBT and 4-CBT were measured from the mixture of SERS ID_[4BBT]_ attached the TG beads and SERS ID_[4CBT]_ attached the TG beads after treatment with 3 wt% BSA containing PBS solution or with 1 wt% Tween 20 containing PBS solution. As shown in Fig. 4b,c, the SERS intensities from representative peaks (488 cm^−1^ for 4-BBT and 539 cm^−1^ for 4-CBT) showed only the original codes. The result indicates that the original encoding for each SERS ID remained intact even after the treatment with the solution containing surfactants, showing no signs of cross-contamination to any significant extent.

Prior to OBOC screening assay, the possibility of signal interference from amino acids was verified. Twenty types of amino acids were coupled on the TG beads, and Raman spectra from the TG beads were measured (Figure S8). No significant additional Raman peaks were observed from any type of amino acid-loaded TG beads. It was because the normal Raman signal intensities of the amino acids were not strong enough to be detected, indicating that there was no signal interference from amino acids in the SERS encoding.

**Figure 5:**
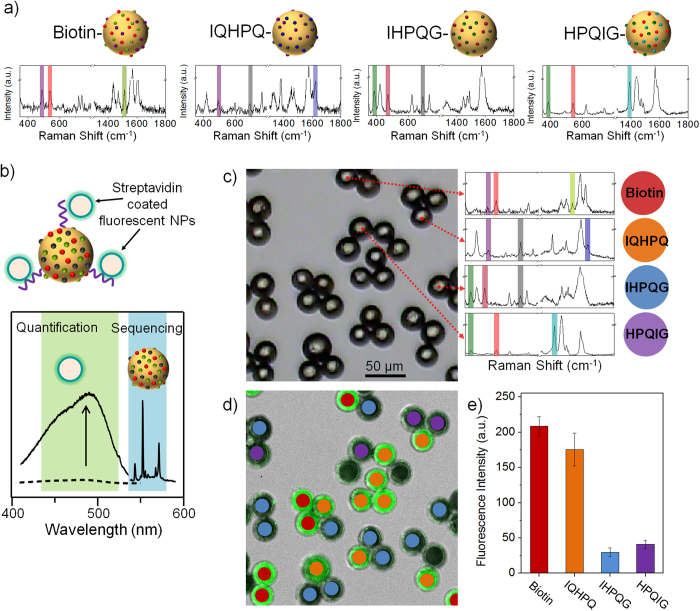
SERS barcoding and screening of solid-phase peptides library. **a**) Model penta-peptides containing HPQ and biotin-loaded TentaGel (TG) beads and their corresponding SERS barcodes using SERS IDs combinations. **b**) Schematic diagram of lead peptides with high binding affinity against streptavidin-loaded TG beads and the identification process based on a fluorescence signal for binding affinity quantification and SERS barcodes for peptide sequencing. **c**) Optical image of TG bead mixtures after the streptavidin-binding assay, and SERS barcodes from the TG beads. **d**) Identification of bio-ligands through decoding of their SERS barcodes. **e**) Histogram for fluorescence signal variation of streptavidin-coated fluorescent nanoparticles binding to TG beads.

Next, the OBOC protein-binding assay was demonstrated (streptavidin was used for proof-of-principle) to explore the strength of the SERS ID-based barcodes in the bioassay. Three kinds of HPQ-containing penta-peptides (IQHPQ, IHPQG, and HPQIG), and biotin as a positive control, were synthesized on photolabile linker-loaded TG beads. Full peptide sequences were synthesized in parallel via standard fluorenylmethyloxycarbonyl (Fmoc) chemistry, and the side chain protection groups were removed by treating with strong acid (95% trifluoroacetic acid) for 1 h. For quality control, the peptide-beads were photocleaved using ultraviolet irradiation, and were analyzed by mass spectroscopy (MS) (Figure S9). Based on the MS data, all the peptides showed clear peaks corresponding to their masses without any significant impurity peaks. Since HPQ containing penta-peptides have been known to have different affinities to streptavidin^1^,^46^,^47^, 4 different bio-ligands, biotin, IQHPQ, IHPQG, and HPQIG, on the TG beads were encoded with combinations of SERS IDs; SERS IDs_[4BBT], [4CBT], and [5PHTT]_, SERS IDs_[4BBT], [BT], and [2NT]_, SERS IDs_[4FBT], [2CBT], and [BT]_, and SERS IDs_[4FBT], [4CBT], and [4ATP]_, respectively, as shown in Fig. 5a. The distinct peaks corresponding to the SERS IDs were completely detected and deconvoluted as barcode presentations with the processing algorithms. The 4 different ligand-loaded TG beads with the corresponding SERS IDs were then mixed together and incubated with streptavidin-coated fluorescent NPs (SA-F-NPs, SPHERO™ streptavidin-coated blue particles, ~400 nm in diameter) for 30 min. Before SERS decoding, fluorescence signals were measured by a confocal laser scanning microscopy. To identify lead peptides which strongly bound to streptavidin, the SERS barcodes of the TG beads was read in the same region of fluorescence measurement using micro-Raman spectroscopy. The fluorescence signals indicate the affinities of peptides against streptavidin, while the SERS barcodes correspond to the peptide sequences or biotin (Fig. 5b). The TG beads were identified from the SERS barcodes to the corresponding peptide sequences or biotin and the 4 kinds of SERS barcodes from representative TG beads were shown in Fig. 5c and Figure S10. Figure. 5d shows a fluorescence image of the SA-F-NP-treated TG beads, and Fig. 5e summarizes the semi-quantitative results of the binding affinity (Fluorescence signal intensities of individual TG bead are shown in Figure S11). The results show that the peptide with the highest binding affinity to streptavidin was IQHPQ. The binding affinity was comparable to that of biotin, consistent with the previous reports^46^,^47^. In contrast, low fluorescence signals were detected for weak binding peptide and for the control peptide group, indicating high specificity and a low level of non-specific protein binding. These findings suggest that the SERS barcoding method can be used to screen biomolecules and has great potential for multiplexed bioassays.

In conclusion, we developed a new class of simple SERS barcoding technology based on SERS active NPs (SERS nano-identifier, SERS ID) for screening of an OBOC library. The barcoding method exhibited high stability and reliability under bioassay conditions - swelling solvent, blocking solution, and washing solution, which was due to the bridges that were formed between the SERS IDs and the entangled chain of the TG beads. The SERS barcodes have great scalability and encoding capacity. By incorporating a combination of SERS IDs, at least one million SERS barcodes could be generated in theory without problematical processes to control intensity levels. As a proof-of-concept experiment, we demonstrated an OBOC screening platform for quantitative profiling of streptavidin-binding specificity. The SERS barcoding technology offers a great promise in the field for screening OBOC libraries and in the applications for drug discovery.

## Methods

### General Information

Tetraethylorthosilicate (TEOS), 3-mercaptopropyl trimethoxysilane (MPTS), silver nitrate (AgNO_3_, 99.999+%), octylamine (OA), sodium silicate aqueous solution (~26.5%), *N, N*-diisopropylethylamine (DIPEA), ethylene glycol (EG), 2-amino-4-chlorobenzenethiol (2-ACBT), 4-azidophenacyl bromide (4-APB), 2-amino-4-(trifluoromethyl)benzenethiol (2-ATFT), 4-aminothiophenol (4-ATP), 3-amino-1,2,4-triazole-5-thiol (3-ATT), 2-bromobenzenethiol (2-BBT), 4-bromobenzenethiol (4-BBT), benzylmercaptane (BMT), benzenethiol (BT), benzyl disulfide (BZDSF), 3-cyanobenzoic acid (3-CBA), 4-cyanobenzylaldehyde (4-CBAL), 2-chlorobenzenethiol (2-CBT), 4-chlorobenzenthiol (4-CBT), 3,4-dichlorobenzenethiol (3,4-DCT), 3,5-dichlorobenzenethiol (3,5-DCT), 3,4-dimethoxythiophenol (3, 4-DMOBT), 2,5-dimethoxythiophenol (2,5-DMOBT), 3,4-dimethylbenzenthiol (3,4-DMT), 3,5-dimethylbenzenthiol (3,5-DMT), 2-fluorobenzenethiol (2-FBT), 4-fluorothiophenol (4-FBT), 4-isopropylbenzenethiol (4-IBT), 3-mercaptobenzoic acid (3-MBA), 4-mercaptobenzoic acid (4-MBA), 2-mercaptobenzimidazole (2-MBI), 2-mercapto-5-methylbenzimidazole (2-MMBI), 2-mercapto-1-methylimidazole (2-MMI), 2-mercapto-6-methylpyridine (2-MMP), 4-methoxybenzenethiol (4-MOBT), 2-mercaptopyrimidine (2-MPY), 4-mercaptotoluene (4-MT), 4-nitrophenyl disulphide (4-NPDSF), 2-naphthalenethiol (2-NT), pentachlorothiophenol (PCTP), 5-phenyl-1H-1,2,4-triazole-3-thiol (5-PHTT), phenyl isothiocyanate (PITC), 5-(4-pyridyl)-1,3,4-oxadiazole-2-thiol (5-PODAT), 4-(pyridin-4-yl) pyridine (4-PPD), 1-phenyltetrazole-5-thiol (1-PTET), 2-quinolinethiol (2-QT), 2-thiazoline-2-thiol (2-TAT), 1H-1,2,4,-triazole-3-thiol (1H-TAT), 2-thiouracil (2-TU), (+)-biotin *N*-hydroxysuccinimide ester, triisopropylsilane (TIPS), bovine serum albumin (BSA), and polyoxyethylene sorbitan monolaurate (Tween 20) were purchased from Sigma-Aldrich (St. Louis, MO, USA). Ammonium hydroxide (NH_4_OH, 27%), absolute ethanol (99.9%), ethanol (95%), methanol, dichloromethane (DCM), *N*-methylpyrrolidone (NMP), piperidine, and trifluoroacetic acid (TFA) were purchased from Daejung (Busan, South Korea). TentaGel (TG) microbeads (0.22 mmol NH_2_/g, 35 μm) were purchased from Rapp Polymere (Tübingen, Germany). Fluorenylmethyloxycarbonyl (Fmoc)-amino acids, 1-hydroxybenzotriazole (HOBt), and (1H-benzotriazol-1-yloxy) [tris(dimethylamino)] phosphonium hexafluorophosphate (BOP) were purchased from Bead Tech Inc. (Seoul, South Korea). Fmoc-photolabile linker (Fmoc-PLL) was purchased by Advanced ChemTech (Louisville, KY, USA). All reagents were used without further purification. Deionized (DI) water was used for all experiments.

### Fabrication of SERS nano-identifiers (SERS IDs)

TEOS (1.6 mL) was dissolved in 43 mL of absolute ethanol containing NH_4_OH (7.5 *v/v* %), and vigorously stirred for 20 h at 25 °C. The resulting silica nanoparticles (NPs) were centrifuged and washed with ethanol several times to remove the excess reagents. These silica NPs were then functionalized with a thiol group. Silica NPs (300 mg) were dispersed in 6 mL of ethanol containing 300 μL of MPTS and 60 μL of aqueous NH_4_OH (27%). After the mixture was stirred for 6 h at 25 °C, the MPTS-treated silica NPs were centrifuged and washed with ethanol several times. For coating of Ag NPs, 50 mg of MPTS-treated silica NPs was thoroughly dispersed in 50 mL of an AgNO_3_ solution (3 mM in ethylene glycol). A volume of 41.3 μL of octylamine was then rapidly added to the dispersed MPTS-treated silica NPs. The resulting dispersion was stirred for 1 h at 25 °C. Afterwards, the resulting Ag-coated silica NPs were centrifuged and washed with ethanol several times for purification.

Next, 1 mL of Raman label compound (25 mM in ethanol) was added to 10 mg of the Ag-coated silica NPs. The resulting dispersion was shaken for 1 h at 25 °C. The Raman label compound-coded Ag-coated silica NPs were centrifuged and washed with ethanol 2 times. To encapsulate the Ag-coated silica NPs with a silica shell, the Ag-coated silica NPs were dispersed in 15 mL of dilute sodium silicate aqueous solution (0.036 *wt*% SiO_2_). The dispersion was stirred with a magnetic stir bar for 15 h at 25 °C. Ethanol (60 mL) was added to the reaction mixture while mixing vigorously with a magnetic stirring bar, and then, the dispersion was stirred for an additional 3 h to form a thin silica shell. Finally, 250 μL of aqueous NH_4_OH (27%) and 30 μL of TEOS were added to the reaction mixture, and it was stirred for 24 h at 25 °C. The resulting SERS IDs were centrifuged and washed with ethanol several times. When compared with other SERS NPs, the Ag NPs embedded-silica based SERS IDs have many advantages such as easy handling, reproducibility and high sensitivity of the signals.

### Synthesis of solid-phase HPQ-containing peptides

The peptides were synthesized on Fmoc-PLL-TentaGel (50 mg, Fmoc-PLL-TG) using conventional Fmoc chemistry. A 20% piperidine/NMP solution was used to remove the Fmoc groups. After removing the Fmoc group, The TG beads were treated with pre-activated Fmoc-amino acid solution that was prepared with Fmoc-amino acid (3 equiv.), BOP (3 equiv., 14.6 mg), HOBt (3 equiv., 4.4 mg), and DIPEA (5 equiv., 9.58 μL) in NMP (2 mL), for 1 h at 25 °C. The amino acid-loaded resin was washed alternately with NMP (×3), DCM (×3), and methanol (×3). After complete peptide synthesis, the terminal amine group was capped with an acetyl group, and side chain protection groups were cleaved with TFA/TIPS/H_2_O (95:2.5:2.5) for 1 h, followed by five washes with NMP.

### Encoding bio-ligands with SERS IDs on TG beads

After the peptide syntheses were completed, the TG microbeads were encoded with a combination of several types of SERS IDs that were physically adsorbed on the microbead surface. Because there are differences in the enhancement effect of each Raman label compound on Ag NPs inside of SERS ID, the amounts of SERS IDs in the mixture were determined from the ratio of the peak intensities for each SERS ID. The determined amounts of each SERS ID are as follows: 8.1 μg for 4-FBT, 32.8 μg for 4-IBT, 17.0 μg for 2-CBT, 7.7 μg for 4-BBT, 11.4 μg for 4-CBT, 10 μg for 3,4-DCT, 2 μg for 4-ATP, 10 μg for 5-PHTT, and 12 μg for 2-NT. The SERS ID mixture (final concentration: ca. 1 wt% to TG bead) corresponding to each peptide was added to the TG bead suspension. The resulting mixture was shaken for 30 min at room temperature. The SERS IDs that were not absorbed were removed by washing with ethanol and vacuum filtration.

### Streptavidin binding reaction and analysis of fluorescence signal

An equal amounts of HPQ-penta peptide TG beads and a biotin-TG beads mixture (10 mg) was incubated with 10 μL of streptavidin-coated fluorescent NPs (SA-F-NPs, 1.0% w/v, SPHERO™ streptavidin-coated blue particles, ~400 nm in diameter) for 30 min. Then, the resulting TG beads were washed with PBS solution (×3), DI water (×3), and vacuum filtration.

Fluorescence images of TG beads were obtained by a confocal laser-scanning microscope (SP8 X STED, Leica; Germany) with an ultraviolet emission line (405 nm) and detection in the 523 ± 75 nm channel. The fluorescence intensities of the TG beads were analyzed with the Leica Application Suite Advanced Fluorescence software (Leica; Germany)

### SERS measurement from SERS ID-coded TG beads

To characterize the SERS IDs, SERS measurements were performed using a confocal micro-Raman system (JY-Horiba, LabRam 300) equipped with an optical microscope (Olympus, BX41). The SERS signals were collected using a ×50 (Olympus, 0.50 NA) and ×100 objective lens (Olympus, 0.90 NA) in a back-scattering geometry and detected using a spectrometer equipped with a thermoelectrically-cooled CCD detector. The 532 nm line of a diode-pumped solid-state laser (CrystaLaser, CL532-100-S) was used as an excitation source for Raman measurements. The laser power at the sample was 2.7 mW with the ×50 objective lens, and 1.0 mW with the ×100 objective lens. For identification of encoded SERS IDs, the SERS spectra were acquired by point-by-point mapping using a ×50 objective lens with a 10 s accumulation time and 1 μm beam diameter.

### Assignment of representative SERS peaks for each SERS ID

One SERS peak with relatively high S/N ratios and little overlap with the peaks of the other SERS IDs is selected as barcode signal for each SERS ID (Fig. 2). By combination of these representative peaks of SERS IDs, various encoding barcodes are generated. The barcodes are represented as binary system, by assigning each selected peak to represent ON (1) values while others OFF (0) values, at corresponding position in the spectral range (Fig. 3).

### Decoding method of the SERS spectra from SERS ID-coded TG beads

To systematically extract barcode presentations by analyzing the obtained SERS spectra from the encoded beads, we designed the automatically decoding algorithm using MATLAB® (MathWorks, Inc.; Natick, MA, USA) as follows. First, we input data about assigned representative SERS peaks and certain cut-off values for each SERS ID, for example less than 2 times of noise value is set zero. And, the intensities of representative peak are assigned as a peak height subtracted with a baseline value. The baseline value is assigned by averaging the intensities at the both sides which are 10 cm^−1^ away from representative peak position. If the intensity is larger than the cut-off value, the existence of SERS ID is cleared, or if not, it doesn’t exist. This check-up process is iterated for the whole SERS IDs. After all, the result of existing SERS IDs is displayed in a barcode form. This decoding process is shown as a flow chart in Figure S4.

## Additional Information

**How to cite this article**: Kang, H. *et al.* Direct Identification of On-Bead Peptides Using Surface-Enhanced Raman Spectroscopic Barcoding System for High-Throughput Bioanalysis. *Sci. Rep.* **5**, 10144; doi: 10.1038/srep10144 (2015).

## Supplementary Material

Supplementary Information
