# Supplementary material for: Direct Identification of On-Bead Peptides Using Surface-Enhanced Raman Spectroscopic Barcoding System for High-Throughput Bioanalysis
Source: Sci Rep. 2015 May 28;5:10144. doi: 10.1038/srep10144 (PMC4446893; doi:10.1038/srep10144)
Supplement: Supplementary Information [file srep10144-s1.pdf]

Supplementary Information

Direct Identification of On-Bead Peptides Using Surface-Enhanced Raman Spectroscopic Barcoding System for High-Throughput Bioanalysis

Homan Kang,[±,‡,†] Sinyoung Jeong,[#,‡] Yul Koh,[⊥] Myeong Geun Cha,[#] Jin-Kyoung Yang,[§] San Kyeong,[§] Jaehi Kim, [§] Seon-Yeong Kwak,[§,†] Hye-Jin Chang,[#] Hyunmi Lee,[§] Cheolhwan Jeong,[§] Jong-Ho Kim,[‖] Bong-Hyun Jun,[Δ] Yong-Kweon Kim,[⊥] Dae Hong Jeong,*[,±,#], and Yoon-Sik Lee*[,±,§]

[±]Interdisciplinary Program in Nano-Science and Technology, [#]Department of Chemistry Education, [⊥]School of Electrical Engineering and Computer Science, and [§]School of Chemical and Biological Engineering, Seoul National University, Seoul 151-744, Republic of Korea

[‖]Department of Chemical Engineering, Hanyang University, Ansan, 426-791, Republic of Korea

[Δ]Department of Bioscience and Biotechnology, Konkuk University, Seoul 143-701, Republic of Korea

* Email: yslee@snu.ac.kr, jeongdh@snu.ac.kr

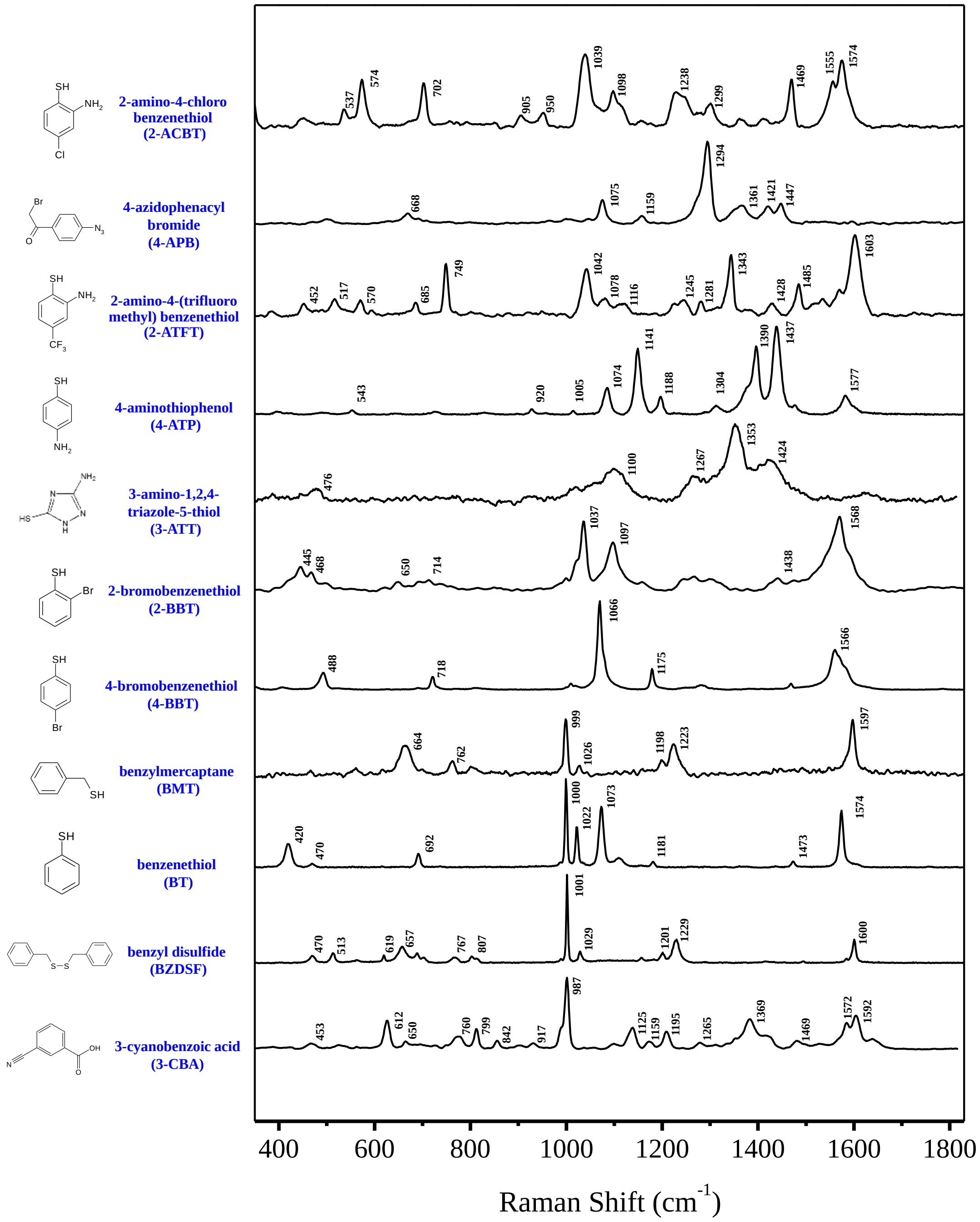

Figure S1. Forty-four kinds of Raman label compounds (chemical structures and names) and their corresponding SERS spectra.

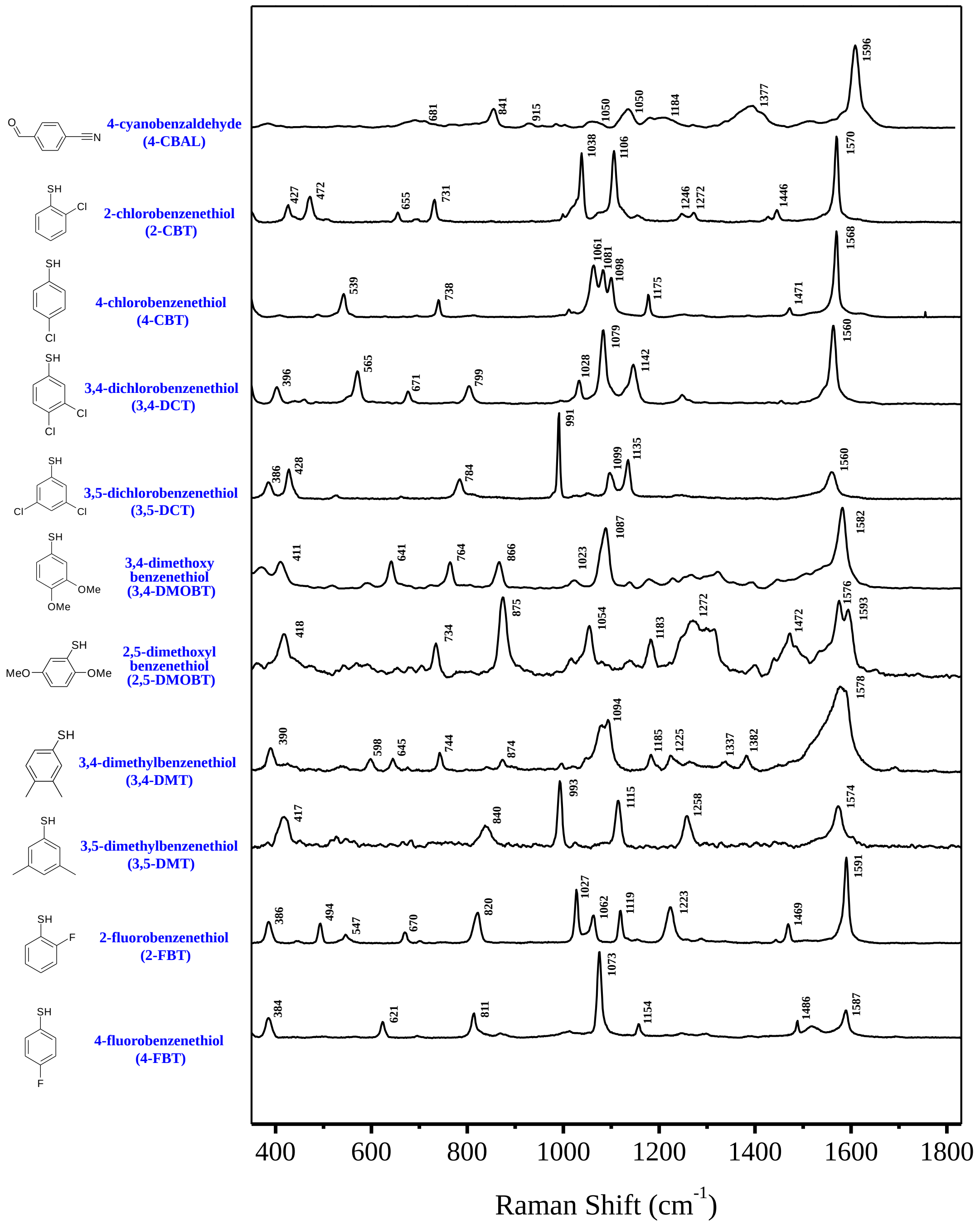

Figure S1. (Continued)

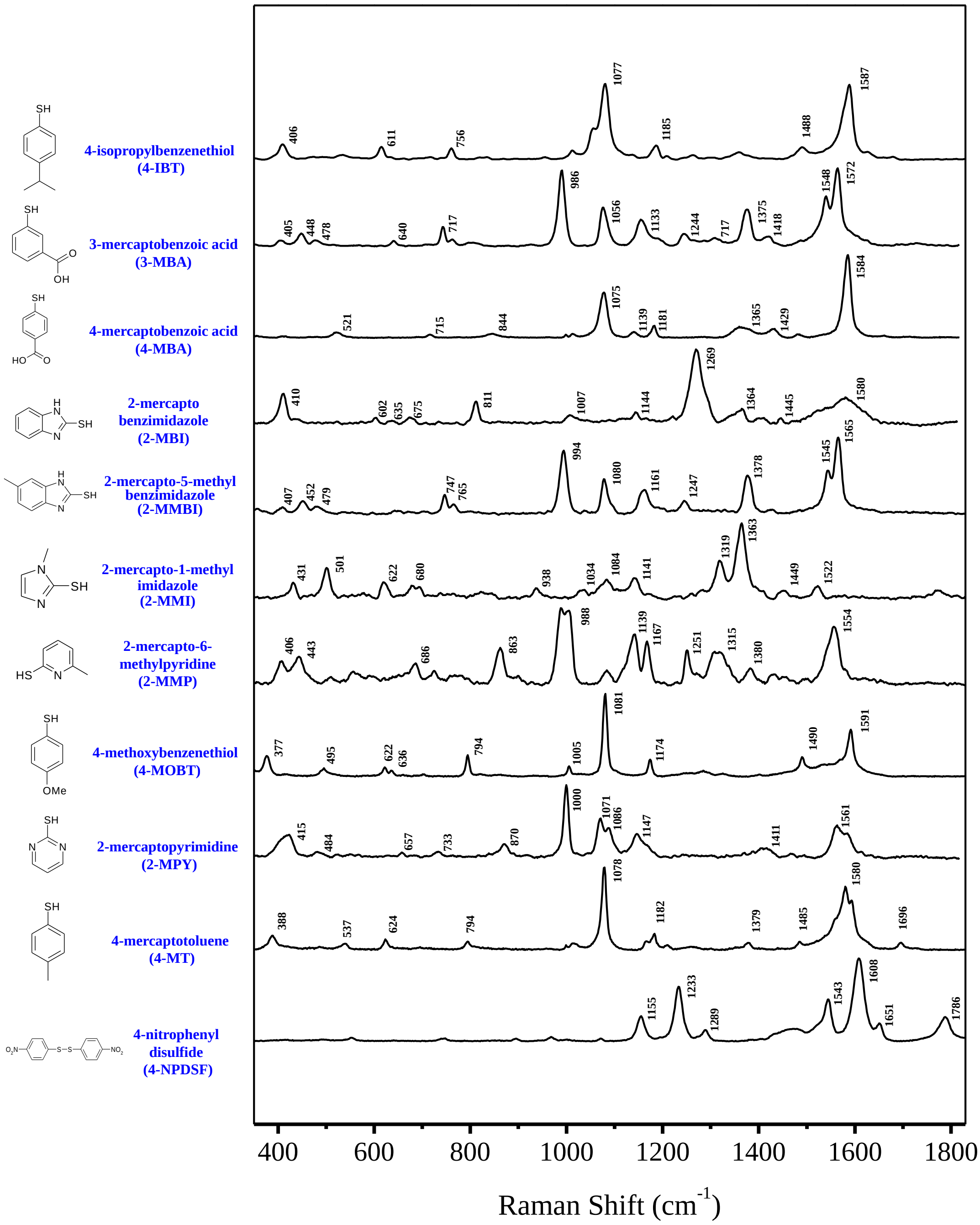

Figure S1. (Continued)

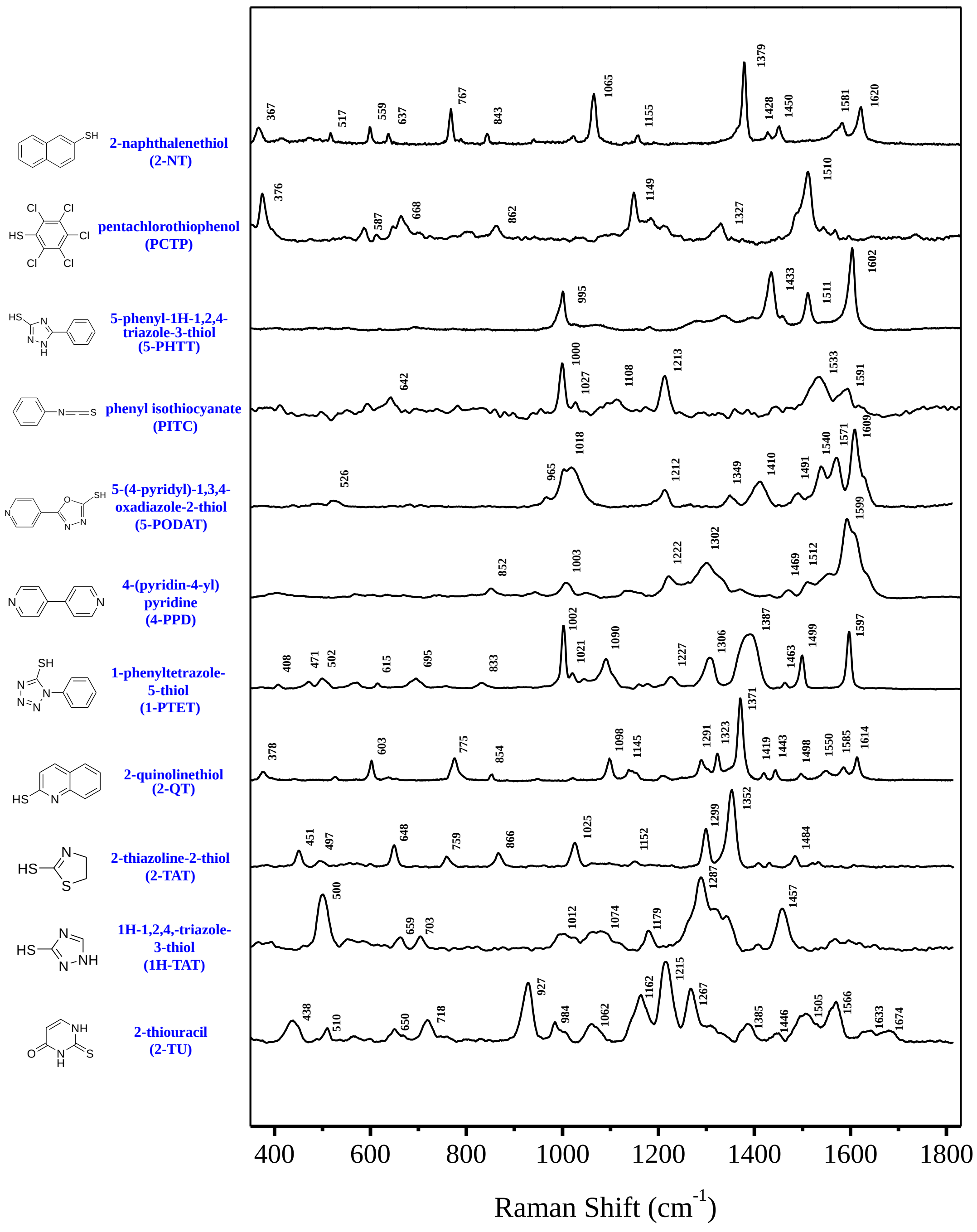

Figure S1. (Continued)

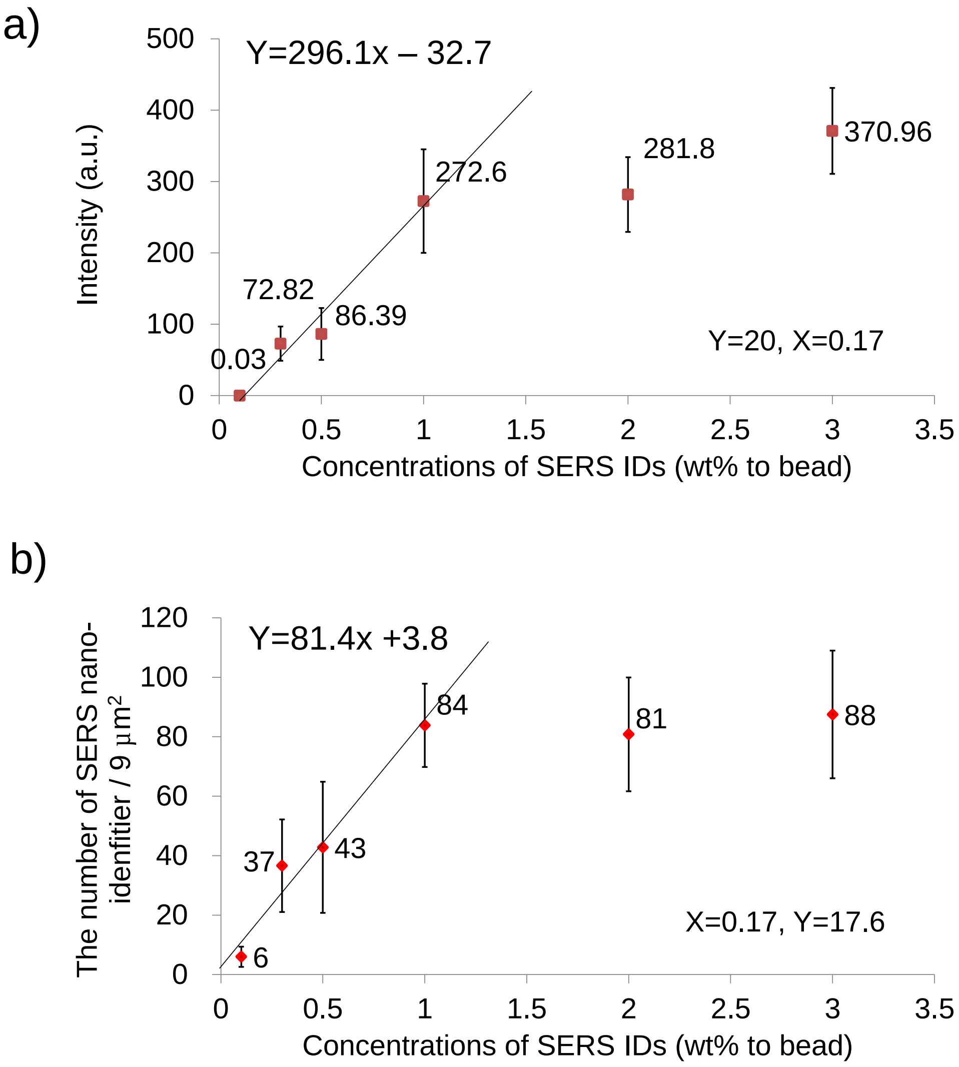

Figure S2. a) Correlation curve between concentrations of SERS nano-identifiers (SERS IDs) and SERS intensities, and b) correlation curve between concentrations and the number of SERS IDs /9 μm^2 TentaGel bead surface. The number of detectable SERS IDs was obtained by fitting the curve with a three-order polynomial, assuming a detectable signal intensity threshold of 20 counts/s.

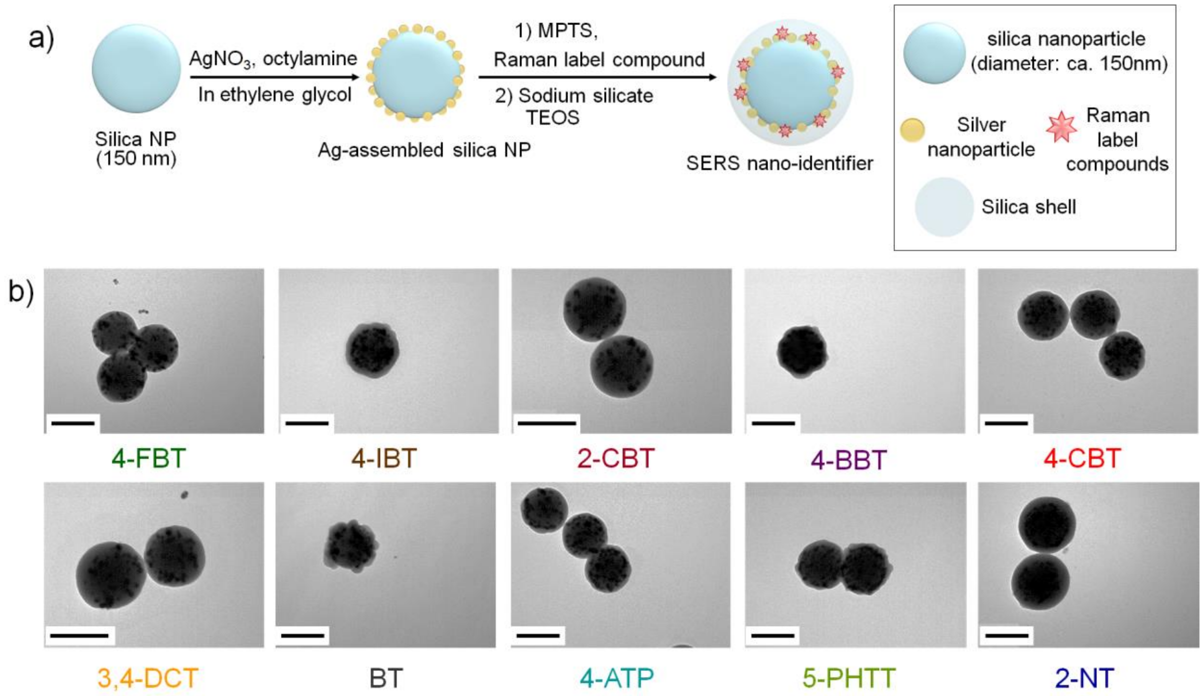

Figure S3. a) Schematic diagram for synthesis of SERS nano-identifiers (SERS IDs) consisting of Ag nanoparticles (NPs) embedded in a silica nanosphere. b) Transmission electron microscopy images of 10 different kinds of SERS IDs. Each scale bar is 100 nm.

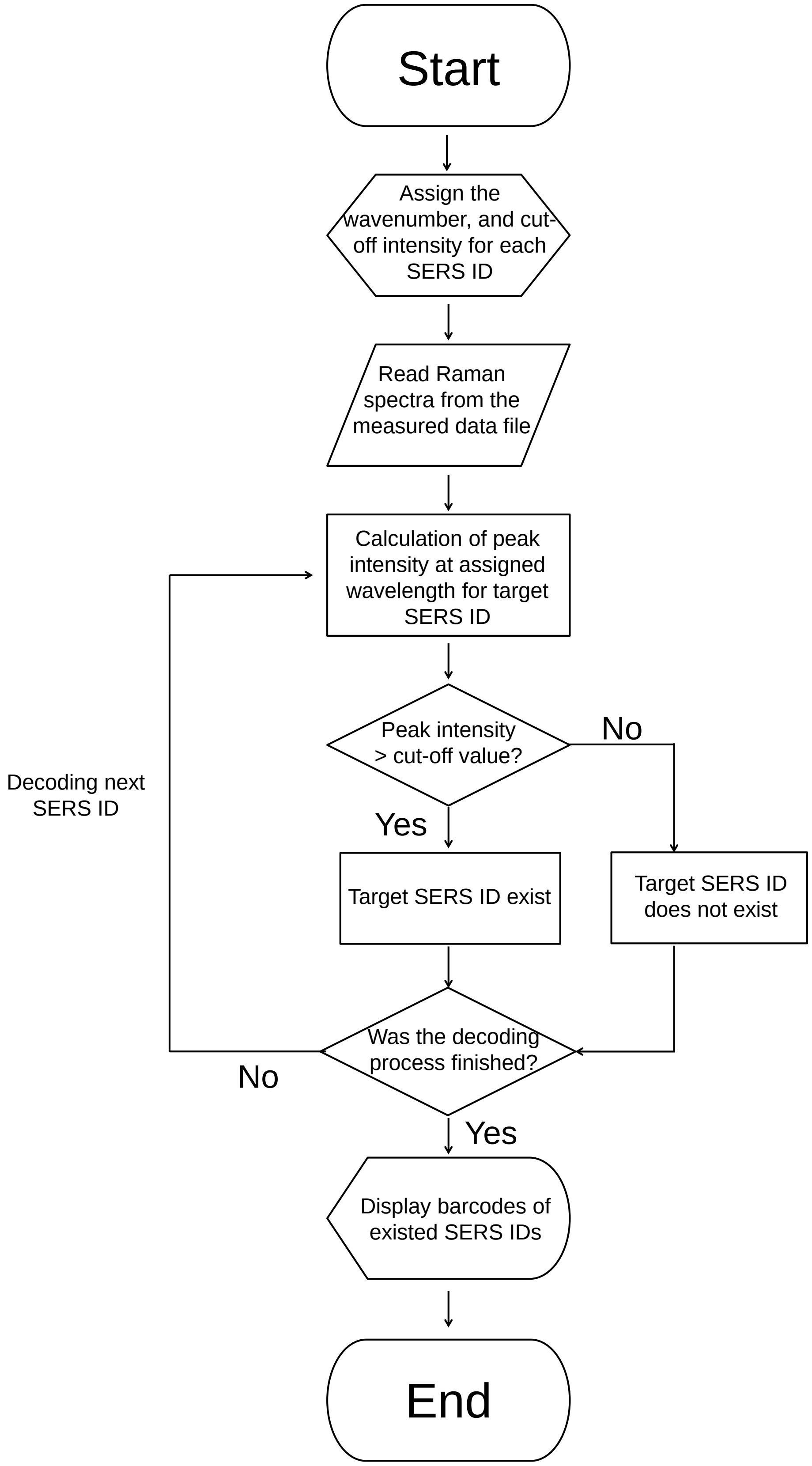

Figure S4. Flowchart of the decoding and barcodes presentation process from the measured SERS spectra of SERS ID encoded TG beads to automatically identify encoded SERS ID on the beads.

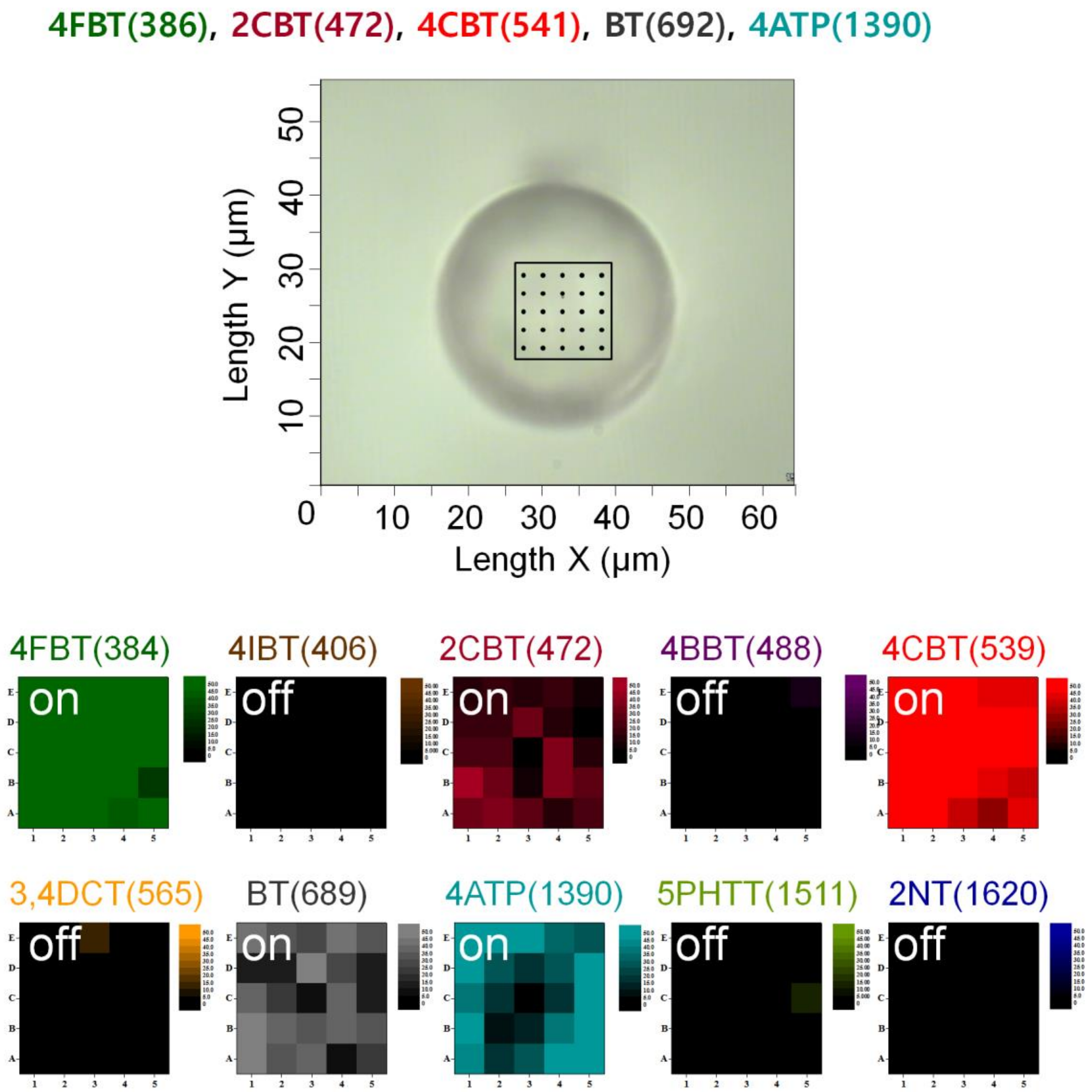

Figure S5. Readings from five combinations of SERS IDs-encoded TentaGel (TG) beads obtained using point-by-point mapping. Optical picture of SERS IDs-encoded TG beads (upper). The bead area (10 × 10 μm) was measured with a 2-μm step size. The SERS intensity maps generated from the SERS IDs-encoded TG beads indicated that the TG bead was encoded with SERS $IDs_{[4\text{-FBT}], [2\text{-CBT}], [4\text{-CBT}], [BT], \text{ and } [4\text{-ATP}]}$.

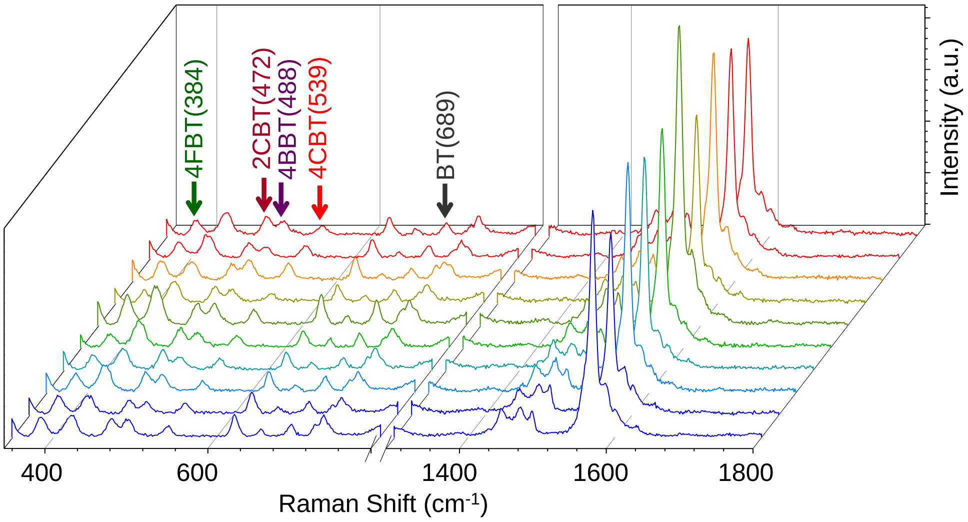

Figure S6. SERS spectra from 10 TentaGel (TG) beads encoded with SERS IDs$_{[4\text{-FBT}], [2\text{-CBT}], [4\text{-BBT}], [4\text{-CBT}], \text{and } [BT]}$. These SERS encoded-TG beads exhibited uniform SERS signals.

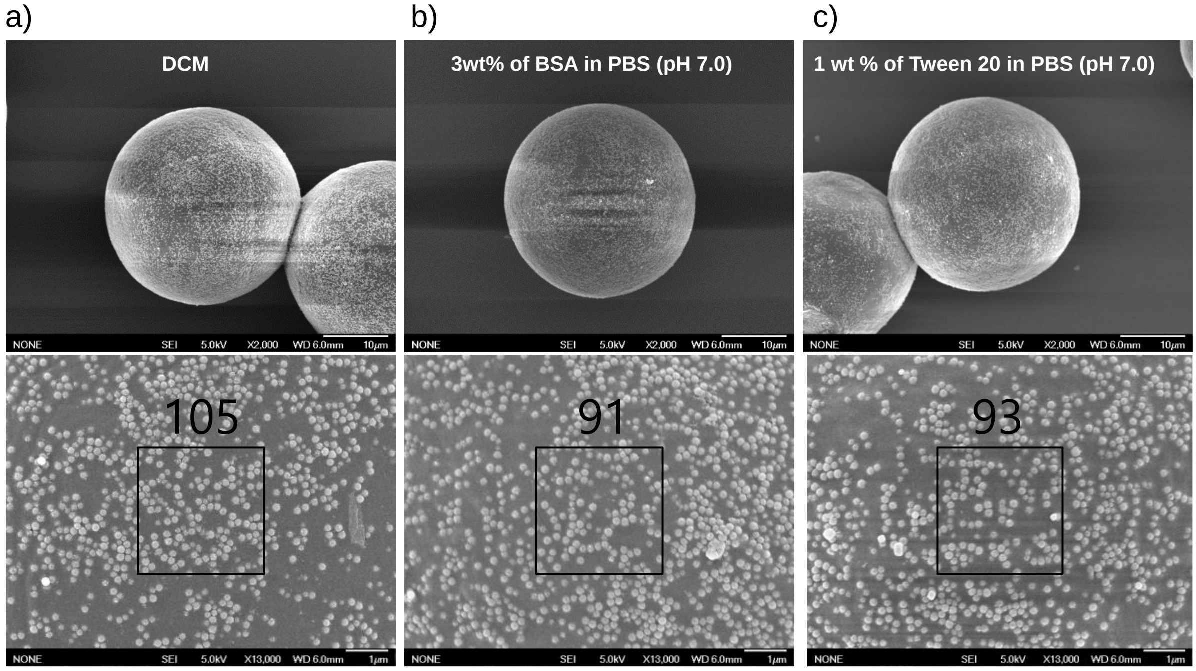

Figure S7. Scanning electron microscopy images of SERS ID-encoded TentaGel beads after treatment with a) swelling solvent (dichloromethane), b) blocking solution (3 % bovine serum albumin [BSA] containing phosphate-buffered saline [PBS]), and c) surfactant containing washing buffer (1 % Tween 20 containing PBS). The lower panel shows high-magnification images of bead surfaces after each treatment condition. The squares indicate 9-μm^2 areas. The numbers indicate the number of SERS IDs /9-μm^2 area.

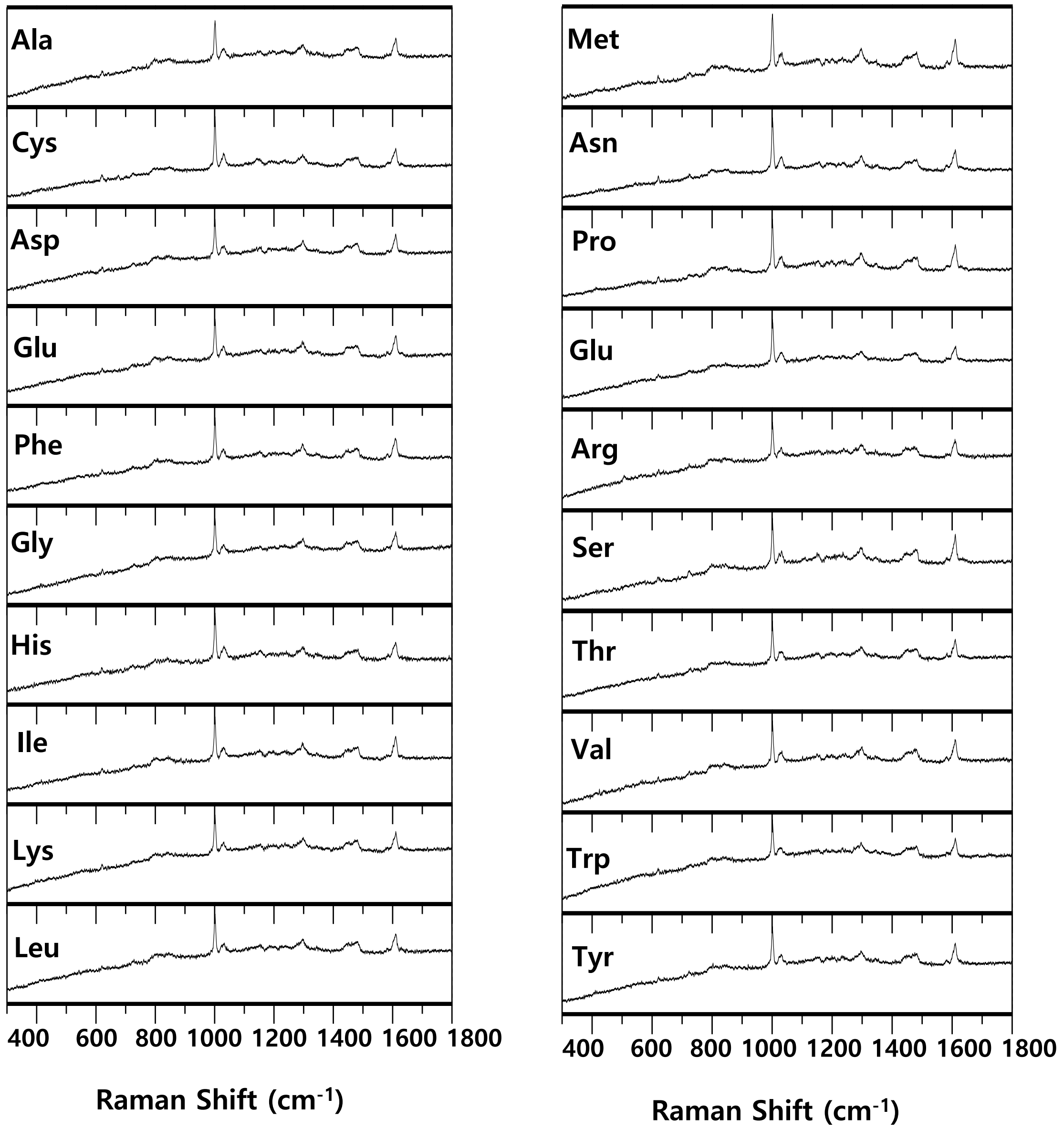

Figure S8. Raman spectra of 20 kinds of natural amino acid-loaded TentaGel beads. No interference Raman peak was observed.

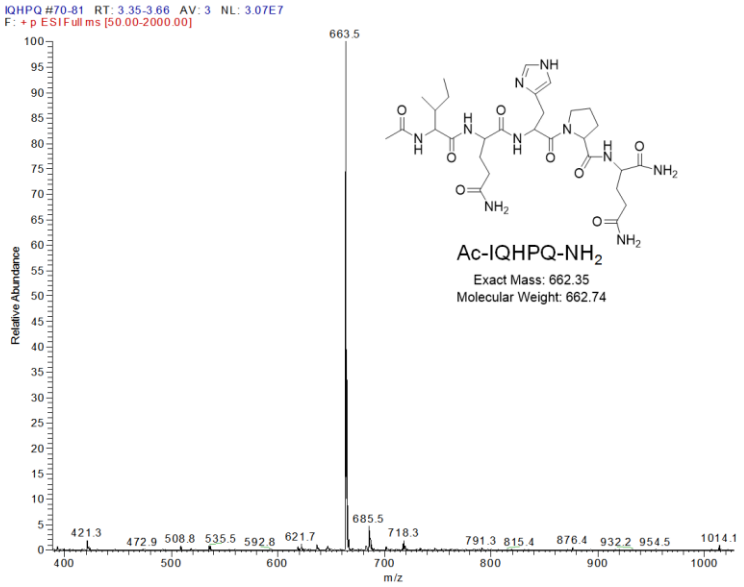

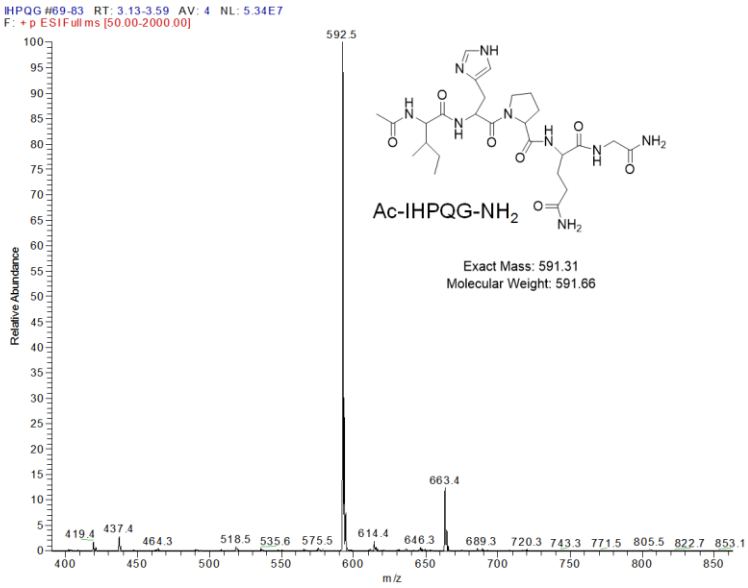

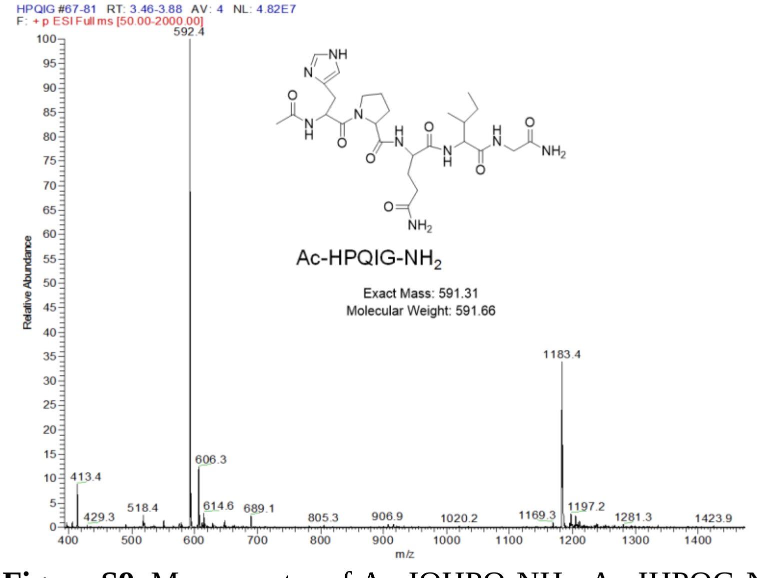

Figure S9. Mass spectra of Ac-IQHPQ-NH_2, Ac-IHPQG-NH_2, and Ac-HPQIG-NH_2.

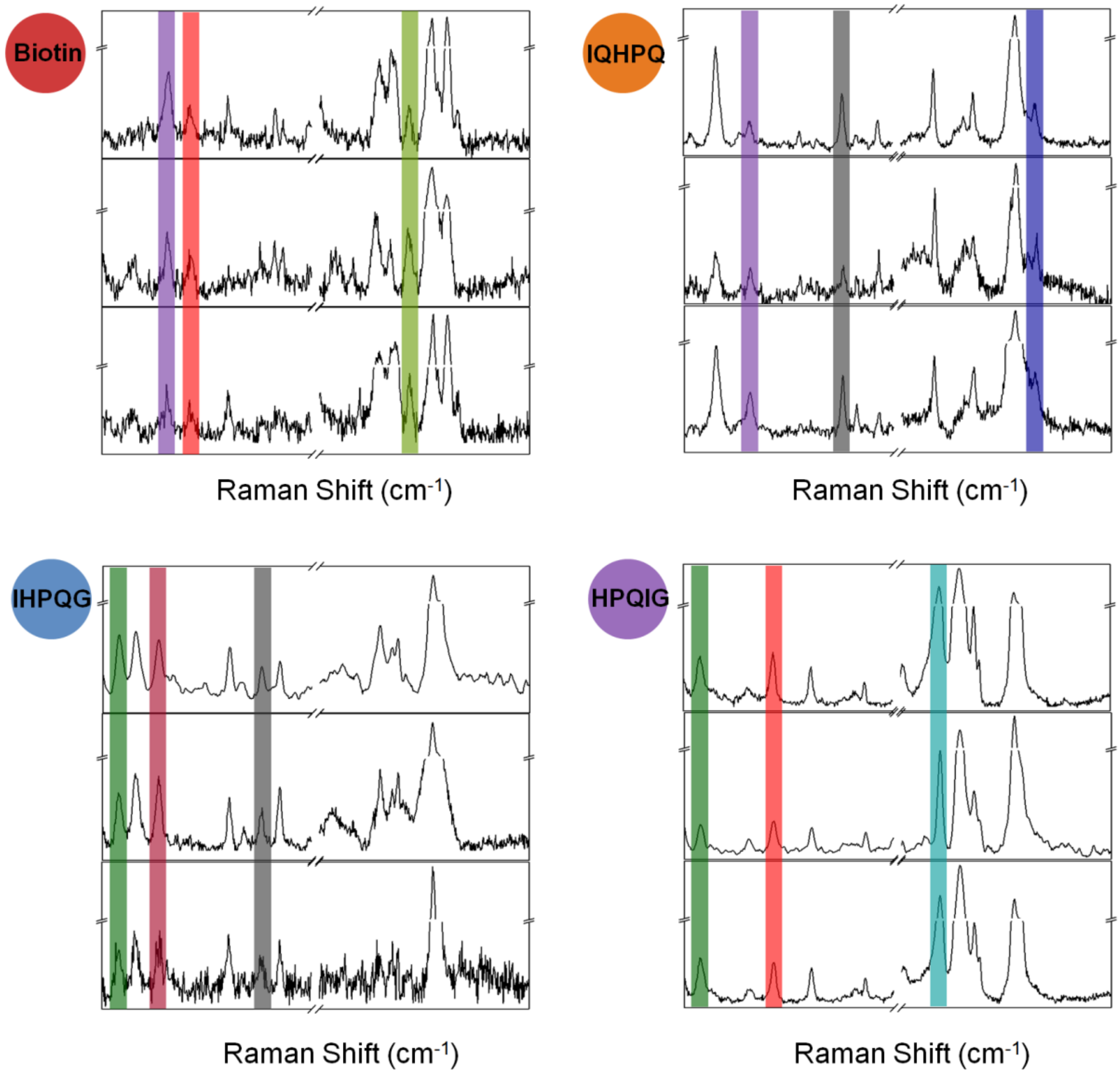

Figure S10. Four kinds of SERS barcodes from TG beads mixtures after streptavidin binding assay.

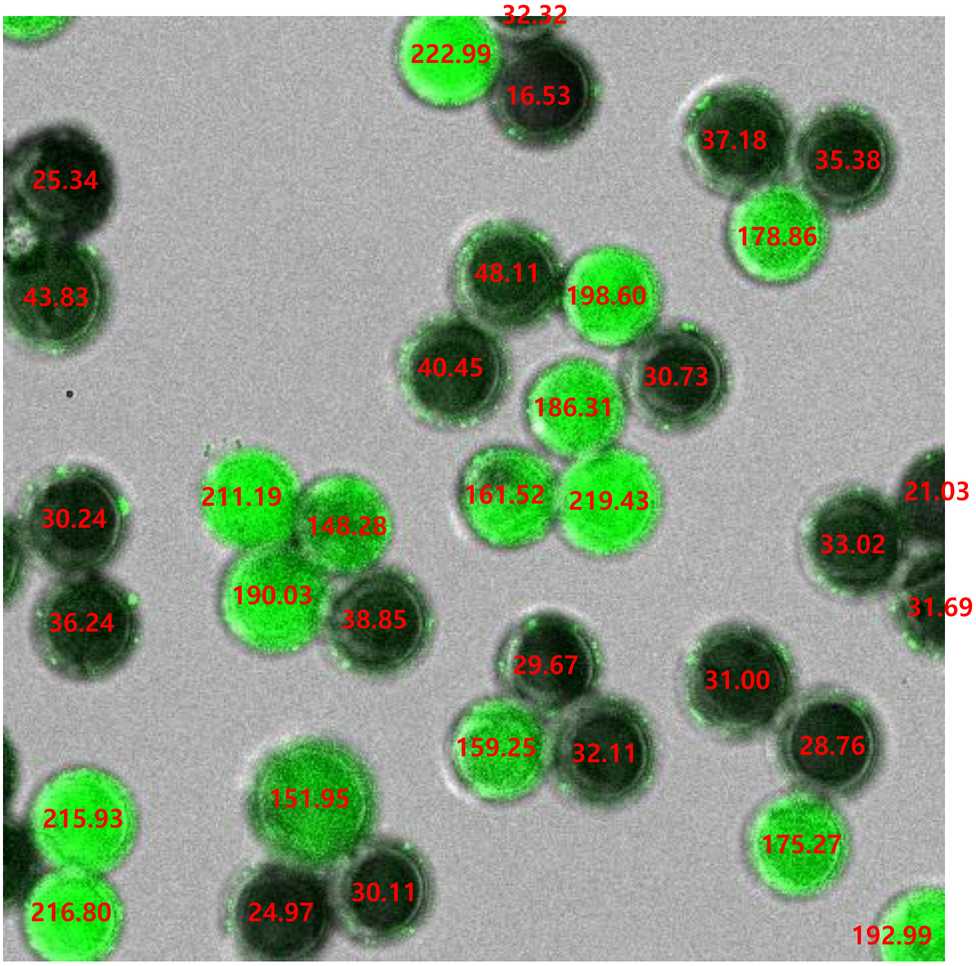

Figure S11. Fluorescence intensity values of ligand loaded-TG beads after the streptavidin-binding assay.
